# Measuring Prosocial Tendencies in Germany: Sources of Validity and Reliablity of the Revised Prosocial Tendency Measure

**DOI:** 10.3389/fpsyg.2017.02119

**Published:** 2017-12-06

**Authors:** Johannes Rodrigues, Natalie Ulrich, Patrick Mussel, Gustavo Carlo, Johannes Hewig

**Affiliations:** ^1^Differential Psychology, Personality Psychology and Psychological Diagnostics, Department of Psychology I, University of Würzburg, Würzburg, Germany; ^2^Differential Psychology, Institute of Psychology, University of Osnabrück, Osnabrück, Germany; ^3^Department of Education and Psychology, Psychological Assessment, Differential and Personality Psychology, Freie Universität Berlin, Berlin, Germany; ^4^Human Development and Family Science, College of Human Environmental Sciences, University of Missouri, Columbia, MO, United States

**Keywords:** prosocial behavior, altruism, validation, measurement invariance, German translation, prosocial tendency measure

## Abstract

The prosocial tendencies measure (PTM; Carlo and Randall, 2002) is a widely used measurement for prosocial tendencies in English speaking participants. This instrument distinguishes between six different types of prosocial tendencies that partly share some common basis, but also can be opposed to each other. To examine these constructs in Germany, a study with 1067 participants was conducted. The study investigated the structure of this German version of the PTM-R via exploratory factor analysis, confirmatory factor analysis, correlations with similar constructs in subsamples as well as via measurement invariance test concerning the original English version. The German translation showed a similar factor structure to the English version in exploratory factor analysis and in confirmatory factor analysis. Measurement invariance was found between the English and German language versions of the PTM and support for the proposed six-factor structure (altruistic, anonymous, compliant, dire, emotional and public prosocial behavior) was also found in confirmatory factor analysis. Furthermore, the expected interrelations of these factors of prosocial behavior tendencies were obtained. Finally, correlations of the prosocial behavior tendencies with validating constructs and behaviors were found. Thus, the findings stress the importance of seeing prosocial behavior not as a single dimension construct, but as a factored construct which now can also be assessed in German speaking participants.

## Introduction

Prosocial behavior is a “voluntary behavior intended to benefit another” (Eisenberg et al., 2006, p. 647). As modern Western society faces many problems arising from a dominance of selfish motives like greed (Mussel et al., 2015) compared to prosocial motives, prosocial behavior and subcategories like altruism are rediscovered as a research topic in science. In neuroscience, one is trying to reveal underlying mechanisms of this behavior (see Weiland et al., 2012; Jiang et al., 2013; Zanon et al., 2014; Rodrigues et al., 2015) often using tasks to measure prosocial behavior, like third party punishment games (e.g., Leliveld et al., 2012) or dictator games (e.g., Forsythe et al., 1994; Eckel and Grossman, 1996; Ben-Ner and Kramer, 2011; Edele et al., 2013). But prosocial behavior can not only be measured via active tasks, but also via questionnaires targeting trait prosocial tendencies. The aim of the present study was to investigate the structure of prosocial tendencies in German-speaking participants via questionnaire. Therefore, a translation and validation of a commonly used measure, the Prosocial Tendencies Measure, Revised (PTM-R; Carlo et al., 2003) was conducted. This would provide German researchers with the opportunity to evaluate the usefulness of this trait measurement of prosocial tendencies with German-speaking populations. Hence, we investigated the internal consistency, the factor structure, and the criterion based sources of validity evidence of a German-language version of the PTM-R with external criterions mentioned below. Moreover, the measurement invariance of the German-language version to the original English-language version of the PTM-R was investigated.

The PTM was developed by Carlo and Randall (2002) measuring prosocial behavior tendencies on six subscales: altruistic (5 items), anonymous prosocial behavior (5 items), compliant prosocial behavior (2 items), dire prosocial behavior (3 items), emotional prosocial behavior (4 items), and public prosocial behavior (4 items). The scales were developed for late adolescents. A revised version, consisting of 25 Items, adding one item to altruistic and one item to emotional prosocial behavior was later published (Carlo et al., 2003). Carlo and Randall (2002) proposed these six subscales of prosocial behavior, because they were not convinced that prosocial behavior is one global behavioral category. The subscales are mostly based on exploratory factor analysis of a literature review done by Carlo and Randall (2002). Although all the subscales reflect a specific form of prosocial behavior, the goal of the questionnaire is to measure prosocial behavioral tendencies. That is, the measure was designed to assess the tendency of individuals to engage in specific forms of prosocial behaviors. The subscales and their correlates are described in the following paragraphs.

First, altruism has been much debated in many disciplines and fields of research. There are many different definitions of altruism. For instance, behavioral altruism has been defined as costly action of benefit to another person (Fehr and Fischbacher, 2003). Carlo and Randall (2002) used the definition made by Eisenberg and colleagues, seeing altruism or altruistic prosocial behavior as “voluntary helping motivated primarily by concern for the needs and welfare of another, often induced by sympathy responding and internalized norms/principles consistent with helping others” (Eisenberg et al., 2006). Also, as the helper is more concerned about the needs of others, costs that can sometimes occur are mentioned (Carlo and Randall, 2002). Some previously made assumptions about the close relation of altruism and empathy (Carlo and Randall, 2002) could not be shown for the PTM-R (Carlo et al., 2003); therefore, empathy is not expected to be directly related to altruism. Previous studies had shown altruism to be negatively related with approval-oriented moral reasoning (Eisenberg et al., 1995) and positively related with the ascription of responsibility, expressing the duty or obligation toward the needs and welfare of others (e.g., Staub, 1978; Batson et al., 1986; Carlo et al., 1991; Schroeder, 1995). For early adolescents, there are additional expected positive correlations with stereotyped and internalized moral reasoning and sympathy (Eisenberg et al., 1995), as well as, weak negative correlations with social desirability (Crowne and Marlowe, 1960). In middle adolescents, Carlo et al. (2003) found an additional weak positive correlation with vocabulary skills and a negative correlation with personal distress. Furthermore, Hardy (2006) showed the predictive value of prosocial moral reasoning for altruism. Finally, Hardy and Carlo (2005) found a significant relation between religiosity and altruism.

Second, compliant prosocial behavior is defined as helping others in response to a verbal or nonverbal request (Carlo and Randall, 2002; Eisenberg et al., 2006), and is expected to occur more frequently than spontaneous helping in the general population (Carlo and Randall, 2002). Carlo et al. (2003) showed a negative correlation between compliant prosocial behavior tendencies and hedonistic prosocial moral reasoning, where acts are only carried out because of the hedonistic gain of the acting person and not due to other reasons (Eisenberg et al., 1995). Additionally, other studies demonstrate positive correlations of compliant prosocial behavior tendencies to empathic accuracy, ascription of responsibility, and sympathy (Carlo et al., 2003). Empathic accuracy in this case means the tendency to take the point of view of another person or simply perspective taking (e.g., Kurdek, 1978; Selman, 1980; Davis, 1983). For early adolescents, there were additional positive relations between this form of prosocial behavior tendencies and internalized moral reasoning (Eisenberg et al., 1995), perspective taking (Davis, 1983) and a negative relation to aggression. In contrast, for middle adolescents, there was a positive relation between compliant prosocial behavior tendencies and needs-oriented moral reasoning (Eisenberg et al., 1995). Further, Hardy and Carlo (2005) showed a positive relation to religiosity, while Hardy (2006) showed correlations to empathy and social desirability (which became weak in later regression analysis) and additionally showed a negative relation to prosocial moral reasoning.

Third, emotional prosocial behavior was conceptualized as helping others under emotionally evocative circumstances. This behavioral tendency was expected to be strongly related to empathy and prosocial moral reasoning (Carlo and Randall, 2002). In the study conducted by Carlo et al. (2003), positive relations to empathic accuracy, ascription of responsibility and internalized prosocial reasoning, as well as negative relations to hedonistic moral reasoning were found. For early adults, there was an additional positive relation with perspective taking, and for middle adolescents with generosity and helpfulness. Other relations found by Hardy (2006) were the predictive value of empathy and prosocial identity as positive predictors to emotional prosocial behavioral tendencies. However, Hardy and Carlo (2005) showed no significant relation between emotional prosocial behavior tendencies and religiosity.

Fourth, public prosocial behavior was seen as helping behavior conducted in front of an audience, motivated at least in part by a desire to gain the approval and respect of others and enhance one's self-esteem (Carlo and Randall, 2002). Carlo and colleagues reported a positive correlation with approval-oriented moral reasoning (Carlo et al., 2003). In addition, Hardy (2006) identified prosocial moral reasoning as a negative predictor of public prosocial behavior tendencies.

Fifth, anonymous prosocial behavior tendencies were defined as helping behaviors where the person receiving the help does not know who offered help (Carlo and Randall, 2002). Carlo et al. (2003) showed a negative correlation between hedonistic prosocial moral reasoning and anonymous prosocial behavior tendencies for middle adolescents, as well as, positive correlations to empathic accuracy and internalized prosocial moral reasoning for early adolescents. Furthermore, Hardy (2006) found that prosocial identity positively predicted anonymous prosocial behavior tendencies in a regression analysis. Finally, Hardy and Carlo (2005) showed a significant relation between anonymous prosocial behaviors tendency and religiosity.

Finally, dire prosocial behavior was defined as helping behavior occurring in crisis or emergency situations, which do not always entail emotionally evocative cues (Carlo and Randall, 2002). Dire prosocial behavior tendencies yielded positive correlations to sympathy, perspective taking and empathic accuracy (Carlo et al., 2003). This study also showed positive relations to needs-oriented and internalized moral reasoning as well as to ascription of responsibility for middle adolescents, and a negative relation to hedonistic moral reasoning for early adolescents (Carlo et al., 2003). Further, Hardy and Carlo (2005) could find no relation between religiosity and dire prosocial behavior tendencies, but Hardy (2006) identified empathy as a meaningful positive predictor of dire prosocial behavior tendencies.

The importance of measuring prosocial behavioral tendencies not as one construct but as different sub-constructs that have different motives, contexts and appearances are discussed by Carlo and colleagues in detail (Carlo and Randall, 2001, 2002; Carlo et al., 2003). In essence, previous research demonstrates that there are specific correlates to specific types of prosocial behaviors (Eisenberg et al., 2006). Furthermore, the interrelations between different forms of prosocial behaviors are often weak or not significant and sometimes negative (Carlo and Randall, 2001).

Taking together the studies using the English-language version of the PTM and PTM-R (Carlo and Randall, 2002; Carlo et al., 2003; Hardy and Carlo, 2005; Hardy, 2006), the expected interrelation of most of the subscales and therefore the constructs mentioned above would be positive and modest. However, no significant relation between public prosocial behavior tendency and other prosocial behavior tendencies, except a strong negative correlation with altruism was found. In addition to the negative correlation with public prosocial behavior tendency, altruism showed no correlation with dire prosocial behavior tendency, maybe indicating the different motives behind these behaviors, with altruism showing also a relation to religiosity and not to empathy, while dire prosocial behavior tendency was correlated with empathy and not with religious motives. Strong positive relations were found between compliant and dire, as well as compliant and emotional prosocial behavior tendencies, and between emotional and dire prosocial behavior tendencies. The aforementioned relation of the subscales dire prosocial behavior and emotional prosocial behavior tendency could be due to the common co-occurrence of these forms of behaviors. Moreover, these forms of prosocial behaviors correlate with empathy and subscales of empathy. Additionally, there seems to be a common motivational basis of emotional, dire and compliant prosocial behavior tendencies because none of these three concepts correlated with hedonistic prosocial reasoning.

These patterns of relations account for often mixed and inconsistent research findings in prosocial behaviors (Carlo and Randall, 2001). Finally, on a conceptual level, scholars have noted that different forms of prosocial behaviors require different sociocognitive and socioemotive skills and that such behaviors demonstrate distinct age-related changes across childhood and adolescence (Eisenberg et al., 2006).

To address these conceptual and empirical issues, Carlo and his colleagues asserted limitations to global, one-dimensional measures of prosocial behaviors and pointed out the need to develop multidimensional measures of prosocial behaviors (Carlo and Randall, 2001, 2002; Carlo et al., 2003). One problem of the global prosocial behavioral measures is that such instruments do not adequately capture the complexity of prosocial behaviors and development (see Eisenberg et al., 1981; Carlo and Randall, 2001, 2002). This approach can also attenuate relations between prosocial behaviors and theoretically relevant constructs. Six commonly-studied forms of prosocial behavioral tendencies were selected to be included in the PTM. The PTM is not designed to assess all possible types of prosocial behavior and other forms of prosocial behaviors exist (e.g. “reciprocal prosocial behavior” is not assessed directly with this scale). However, the six types included in the PTM are theoretically important forms of prosocial behaviors that cut across distinct motives and contexts of prosocial behaviors. Therefore, the German-language version of the PTM-R is designed to assess the six forms of prosocial behavioral tendencies of the original version of the PTM-R.

### Hypotheses

A first goal of the present study was to translate and to validate the translation of the PTM-R into German. Therefore, the factor structure of the German version of the PTM-R was analyzed using exploratory and confirmatory factor analysis on the proposed model underlying the PTM (Carlo and Randall, 2002) and the PTM-R (Carlo et al., 2003). Additionally, we investigated the intercorrelations of the scales. We expected to find a similar pattern of correlations compared to the original version, as outlined above.

The second goal of the present study was to contribute to our knowledge about the nomological net of altruism and its subscales. Particularly, we investigated the relations between altruism and several theoretically-relevant and thus far understudied concepts.

Specifically, empathy, cognitive sensitivity, emotional sensitivity, emotional concern, cognitive concern, social desirability (all assessed in subset 1), positive and negative affect (assessed in subset 2), neuroticism, extraversion, openness, agreeableness, conscientiousness (all assessed in subset 2 and subset 3) as well as their aspects (assessed in subset 3) were examined as correlates of the PTM-R subscales to evaluate sources of validity evidence. The rational to choose these constructs are very diverse. The reason to choose empathy was the relation of prosocial acts in third person economic games and empathy (e.g., Leliveld et al., 2012). So as we expected a correlation of prosocial behavioral tendencies and empathy, we tried to further disentangle the term empathy into subscales (cognitive sensitivity, emotional sensitivity, emotional concern, cognitive concern), possibly targeting different prosocial behavior tendencies, showing the differences in the underlying motives of different prosocial behavioral tendencies. Trait positive and negative affect was included in the analysis because of the different emotional states that can be induced by the situation that leads to specific types of prosocial behavior (e.g., dire prosocial behavior or emotional prosocial behavior, see Carlo and Randall, 2002; Carlo et al., 2003). Hence if the tendency to experience negative or positive affect more often is given, it may have an influence on some prosocial behavior tendencies. For the big five personality traits, the rational to include extraversion was that one motive behind anonymous prosocial behavior might be that the helper is introverted (see Mukahi et al., 1998). Also for public and emotional prosocial behavior tendencies, we expected a higher extraversion because of the kind of expression management character of both kinds of behavior (e.g., Krämer and Winter, 2008). For similar reasons, neuroticism was included, with an additional positive relation to dire prosocial behavior as the person might have higher empathy for people in distress, as they may also have a higher sensitivity for emotional distress (e.g., Jolliffe and Farrington, 2006). For agreeableness, compliant prosocial behavior and altruism might partly be driven by this personality trait, for a sub-dimension of agreeableness is altruism and another sub-dimension is cooperation (DeYoung et al., 2007). For openness, as one of its sub-dimensions is emotionality or openness for feelings (see DeYoung et al., 2007), emotional prosocial behavior might be correlated to this construct and for conscientiousness, we expected a more negative relation to the prosocial behavior tendencies that are driven by intense situations (e.g., dire, emotional, see Murphy et al., 2013 for interpersonal stress) compared to the more steadily appearing ones (e.g., altruism). Finally, the social desirability scale was included because of the different desirability of the prosocial behavior tendencies. Here, an altruistic way of life is something one might want to express, when thinking of oneself while public prosocial behavior may have a flaw in this perspective, as one is still trying to get the most out of the situation, which is not socially desirable in most cases.

Beyond the specific reason mentioned above, another reason to include these constructs was to identify a possible common basis for different kinds of prosocial behavior tendencies. In an additional attempt to validate a differentiation between the different prosocial behavior tendencies that were assessed with the PTM-R, we tried to assess behavioral measures of altruistic acts via dictator game (see Rodrigues et al., 2015), that should correlate with altruism, and assessed voluntary work, which we hypothesized to be negatively correlated with dire prosocial behavior, as one might do the volunteer work for a longer time and not just in the situation of a crisis. Finally, a measurement invariance test with a sample of European Americans using the English language version of the PTM-R was performed in order to test the factor construct of the PTM-R in Germans.

## Method

### Participants

A total of 1,067 participants (687 women, 380 men, *mean age* = 27.672 years, *SD* = 11.674, *Range* = 18−71) were recruited via online questionnaire. Most of the participants were recruited during a university course from students of the course and filled in an online questionnaire. The questionnaire was presented with SoSci Survey (Leiner, 2014) or was presented on a website, using the generic form processor (Göritz and Birnbaum, 2005) for html surveys (in subset 1). The data acquired via SoSci Survey was included in the analysis if it fulfilled the data quality criteria, consisting of having less than 100 negative points, gained by missing data (weighted by absolute missing data to the question) and an unreasonable short time ({[(median of time in s used for the page)/(time in s used for the page) − 1]/2, limited from 1 to 5}^*^100) for answering questions. For more details about the data quality criteria, see Leiner (2016). More details concerning the subsamples can be found below and in Tables ESM5 and ESM6 (Supplementary Material).

#### Subset 1

For this study, 285 student participants (208 women, 76 men, *mean age* = 22.84, *SD* = 4.052, *Range* = 18–54) filled in an online version of the questionnaires.

#### Subset 2

For this study, 360 participants were recruited and 339 participants (202 women, 128 men, *mean age* = 28.31, *SD* = 12.317, *Range* = 18–71) fulfilled the data quality criteria.

#### Subset 3

In this study, 326 participants were recruited and 294 participants (178 woman, 116 man, *mean age* = 30.07, *SD* = 13.252, *range* = 18–68) fulfilled the data quality criteria. The sample was diverse in terms of age and education, with the mode in students and young workers with academic background.

### Instruments

The main questionnaire used was the translated version of the Prosocial Tendencies Measure (Carlo and Randall, 2002), revised version (PTM-R Carlo et al., 2003) with the six subscales noted above. Sample items and the response scale are available in the Appendix section. Also, demographical data was collected (e.g., age, gender, education). In subset 1, subset 2 and subset 3, additional questionnaires were used to determine the relation to different, yet similar constructs, and the big 5 personality traits (see the description of the subsets for a detailed list of the questionnaires included within each subset). The translation of the PTM-R was made by the authors and it was retranslated to English by one of the authors not knowing the original questionnaire at that time. Additionally, retranslation was made by a German English teacher. The two retranslations showed no substantial differences to the original version of the PTM-R, hence we concluded that the translation properly reflects the conceptual meaning of the original version. To provide a measurement invariance test of the English language and German language versions of the PTM, the German version of the PTM-R was shortened to 23 items to match the same items used by Carlo and Randall (2002). The German version of the PTM-R can be seen in the Supplemental Material and in the Appendix section.

#### Subset 1

The instruments used were the translated version of the PTM-R (Carlo et al., 2003), the E-Skala (Leibetseder et al., 2001), measuring empathy with the subscales cognitive sensitivity, emotional sensitivity, emotional concern, cognitive concern, a translated version of the Crowne-Marlowe-Social-Desirability-Scale (SDS-CM, Crowne and Marlowe, 1960; Lück and Timaeus, 1969) and demographical data (e.g., gender, age and income). Cronbach's alpha and the number of items for every construct can be seen in **Table 3**.

#### Subset 2

The instruments used were the translated version of the PTM-R (Carlo et al., 2003), the NEO–FFI (Costa and McCrae, 1989; Borkenau and Ostendorf, 1993), and the PANAS Scales (Watson et al., 1988; Krohne et al., 1996). Cronbach's alpha of these instruments can be seen in **Table 3**. Also, demographical data was collected (e.g., age, gender, education).

#### Subset 3

The instruments used were the translated version of the PTM-R (Carlo et al., 2003) and the big five aspect scales (BFAS, DeYoung et al., 2007). Cronbach's alpha of the scales and aspects can be seen in Table 3. Also, demographical data was collected (e.g., age, gender, education). Additionally, a one-shot dictator game (e.g., Kahneman et al., 1986; Güth, 1995; Haselhuhn and Mellers, 2005; Mellers et al., 2010) was played with the participants, where the participants were able to decide to distribute an amount of money between themselves and a (fictive) receiver who had no chance of influencing this distribution (for further details on the dictator game, see Rodrigues et al., 2015). Although the participants were not informed whether they would get the money of this one shot dictator trial at that time, and did not get any incentives for this one shot dictator game in the end, the division might still be a valid measure of prosocial behavior, because this kind of fictive decision has also been used in dictator games before and came to similar results than the normal dictator game (Ben-Ner et al., 2008).

### Procedure

Participants had to fill in the online questionnaire and were able to receive a feedback of their personality afterwards. No other studies were following the online study except in subset 1, where according to their results, some of the participants were invited to participate in a second study (Rodrigues et al., 2015).

### Data analytic approach

The means, standard deviations, and ranges for the subscales of the German version of the PTM-R were computed. Item-total correlation, Cronbach's Alpha of the subscales if the item was deleted and endorsement were computed, as well as McDonald's omega and the Bonferroni adjusted correlations of the subscales. Additionally, multivariate ANOVA was used to determine differences in prosocial behavior tendencies for the gender of the participants. The McDonald's omega was computed using the MBESS package version 4.4.1 (Kelly, 2017) for R software (R Core Team, 2016).

An odd-even split of the participants' numbers was made for the data to perform exploratory factor analysis and confirmatory factor analysis on different samples. The exploratory factor analysis was computed with the psych package (Revelle, 2016) for R software (R Core Team, 2016). The criteria to determine the number of factors was parallel analysis (Horn, 1965). The rotation of the factors was oblique to leave out unnecessary restrictions and to investigate the relation of the different subscales.

After performing exploratory factor analysis, Rasch analysis was performed with the eRm package (Mair et al., 2016) for R software (R Core Team, 2016) on the dataset of the exploratory factor analysis in order to determine the item-response curves for the items. The rating scale model was chosen as a default model for every subscale of the PTM, as one rating scale was used to answer all the items of the questionnaire (see Appendix).

Also, confirmatory factor analysis was performed with onyx software (Oertzen et al., 2015). The six-factor solution with the six factors proposed by Carlo and colleagues (Carlo and Randall, 2002; Carlo et al., 2003) was used as the target model. This target model was compared to a one-factor solution. To get detailed information about the model fit, several approximate fit indices (Kline, 2011) provided by onyx software are reported. Akaike information criterion (*AIC*) and bayesian information criterion (*BIC*) for the comparison with the one-factor model. *X*^2^_model_, a fit index that should not be significant, is reported along with Root Mean Square Error of Approximation (*RMSEA*), indicating good fit if *RMSEA* ≤ 0.06 (Hu and Bentler, 1999) and poor fit if *RMSEA* > 0.1 (Browne and Cudeck, 1992), but examining the 90% confidence interval is recommended (Kline, 2011). Also, Bentler Comparative Fit Index (*CFI*) is reported, as well as Tucker-Lewis index indicating good fit the higher they are, with previous recommendations being higher than 0.95 (Bentler, 1990; Kline, 2011). Additionally, Standardized Root Mean Square Residual (*SRMR*), that should be ≤0.08 (Hu and Bentler, 1999) was reported. As onyx software does not report the 90% confidence interval of *RMSEA*, the model was also run with lavaan package (Rosseel, 2012) for R software (R Core Team, 2016). For additional information of selecting the fit indices, see Schweizer (2010).

The measurement invariance test was performed with the software package semTools (semTools Contributors, 2016), comparing the present sample with a size matched sample of college students from the University of Missouri (mean age = 19.25, *SD* = 1.28; 65% female, *N* = 543, sampled pseudo-randomly to 533 with random-seed = 13 from sample in R, in order to prevent a lack of sensitivity for changes in measurement invariance due to unequal sample size, see Chen, 2007) measured with the English version of the PTM. All participants of the matched sample had English as their first language and the English version of the PTM was used to assess the prosocial tendencies. For determining measurement invariance, the recommended Δ-CFI was used, because it is not influenced by model complexity or sample size (Cheung and Rensvold, 2002; Meade et al., 2008). Following Cheung and Rensvold (2002) a Δ-CFI≤0.01 indicates that the null hypothesis of invariance should not be rejected. However, Chen (2007) defines Δ-CFI≤0.01 combined with a change in RSMEA≤0.015 as indicator for measurement invariance for equal sample sizes. Meade et al. (2008) mention Δ-CFI ≤ 0.002 as relevant indicator of measurement invariance *per se*, although they mention that their simulation provided a better fit than real data and that in sample sizes greater than 200 it is probable that any (small) differences between groups are trivial in nature.

#### Subsets

The means, standard deviations, and ranges for the subscales of the German version of the PTM-R were computed for both genders and in total. Cronbach's alpha and McDonald's omega of the subscales of the E-Skala (subset 1), the SDS-CM (subset 1), PANAS Scales (subset 2), NEO-FFI (subset 2), and the BFAS (subset 3) as well as the Bonferroni adjusted correlations of the subscales of the PTM-R, the E-Skala (subset 1), the SDS-CM (subset 1), PANAS Scales (subset 2), NEO-FFI (subset 2) and the BFAS (subset 3) were also computed. Additionally, Bonferroni adjusted correlations between the PTM-R subscales and the amount of money offered in the one-shot dictator game were computed in subset 3.

### Ethics statements

This study was carried out in accordance with the recommendations of “Ethical guidelines, The Association of German Professional Psychologists” (“Berufsethische Richtlinien, Berufsverband Deutscher Psychologinnen und Psychologen”) with written informed consent from all subjects. All subjects gave written informed consent in accordance with the Declaration of Helsinki. The protocol was not approved by any additional ethics committee, for it was simply a translation and combination of different self-report questionnaires in one study, combined with short self-reports or a dictator game. If the participants took part in the dictator game, a cover story was used, but they were told about this deception as soon as the task was over, as it is common practice in psychological experiments.

## Results

The means, standard deviations, and ranges for the subscales of the PTM-R are presented in Table ESM1. Participants scored highest on the altruistic prosocial behavior subscale, followed by compliant prosocial behavior, dire prosocial behavior, emotional prosocial behavior and anonymous prosocial behavior. The lowest scores were observed for public prosocial behavior. Multivariate ANOVA revealed a gender difference, showing a higher score in emotional prosocial behavior [*F*_(1/1065)_ = 67.42, *p* < 0.001, *partial* η^2^ = 0.06], altruism [*F*_(1/1065)_ = 21.9, *p* < 0.001, *partial* η^2^ = 0.02] and compliant prosocial behavior [*F*_(1/1065)_ = 14.36, *p* < 0.001, *partial* η^2^ = 0.01] for women, and a higher score in public prosocial behavior in men [*F*_(1/1065)_ = 47.52, *p* < 0.001, *partial* η^2^ = 0.04].

Correlation of the subscales can be seen in Table 1. As hypothesized, there was a modest interrelation of the subscales with some exceptions. As expected, the public subscale was highly negatively correlated with the altruism subscale and not correlated with dire, anonymous or compliant prosocial behavior. Additionally, there was no significant correlation between altruism and anonymous prosocial behavior, as well as altruism and emotional prosocial behavior. Cronbach's alpha and McDonald's omega can be seen in Table ESM2. Lowest endorsement was in public prosocial behavior and highest endorsement in altruism.

**Table 1:** Correlations of the subscales and the different factors of the German version of the PTM-R.

**Factor/prosocial behavior category**	**Altruism**	**Anonymous**	**Public**	**Emotional**	**Dire**	**Compliant**
Altruism	–	0.04	−0.49	−0.06	0.15	0.19
Anonymous	−0.01	–	−0.01	0.16	0.23	0.06
Public	−0.56^**^	0.07	–	0.17	−0.02	−0.03
Emotional	−0.12^**^	0.19^**^	0.22^**^	–	0.40	0.35
Dire	0.01	0.29^**^	0.08	0.43^**^	–	0.30
Compliant	0.09^*^	0.16^**^	0.02	0.31^**^	0.39^**^	–

*Below the middle diagonal the correlation of the subscales are shown, above the diagonal the correlation of the different factors are shown*.

* *p < 0.05*;

** *p < 0.01, Significance levels of the correlations for the subscales have been Bonferroni adjusted. Sample size is 1,067 for the correlations of the subscales and 534 for the factor solutions*.

**Figure 1:**
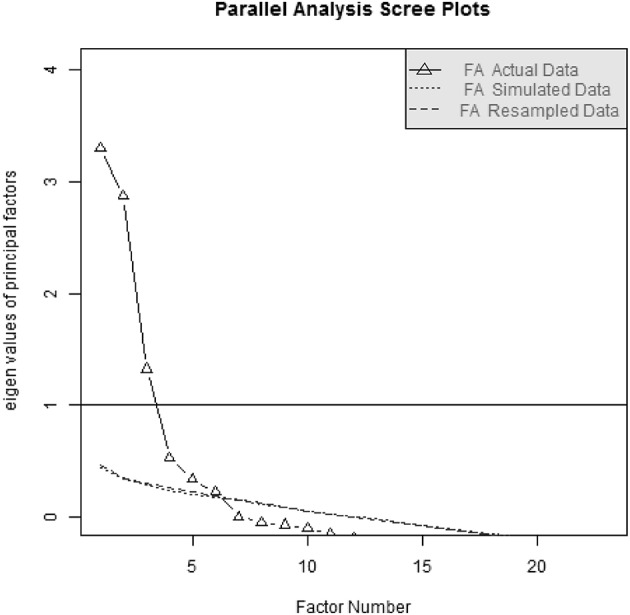
Parallel analysis of the German translation of the PTM-R. The factor solution suggests 6 factors.

The exploratory factor analysis led to a six-factor solution (see Figure 1). The fit of this factor solution was 0.84, the fit of the off diagonal elements was 0.99. In order to verify the six-factor solution and compare with a one-factor solution, also a one-factor solution with fit 0.34 and the fit of the off diagonal elements 0.44 was computed and discarded (see also ESM statistic section, folder R). The factor loadings for the six-factor solution can be seen in Table ESM3. As proposed in the original English version, the same factors were extracted from the translated item pool. Only the Item altruism1 did not load accordingly on its desired factor (see Tabachnick and Fidell, 2007). The interdependence of the different factors can be seen in Table 1. Here, similar to the original English version, we saw the negative relation of the factors altruism and public prosocial behavior (*r* = −0.49), as well as the positive correlations between dire and emotional prosocial behavior (*r* = 0.40), compliant and dire prosocial behavior (*r* = 0.30) and compliant and emotional prosocial behavior (*r* = 0.35).

**Figure 2:**
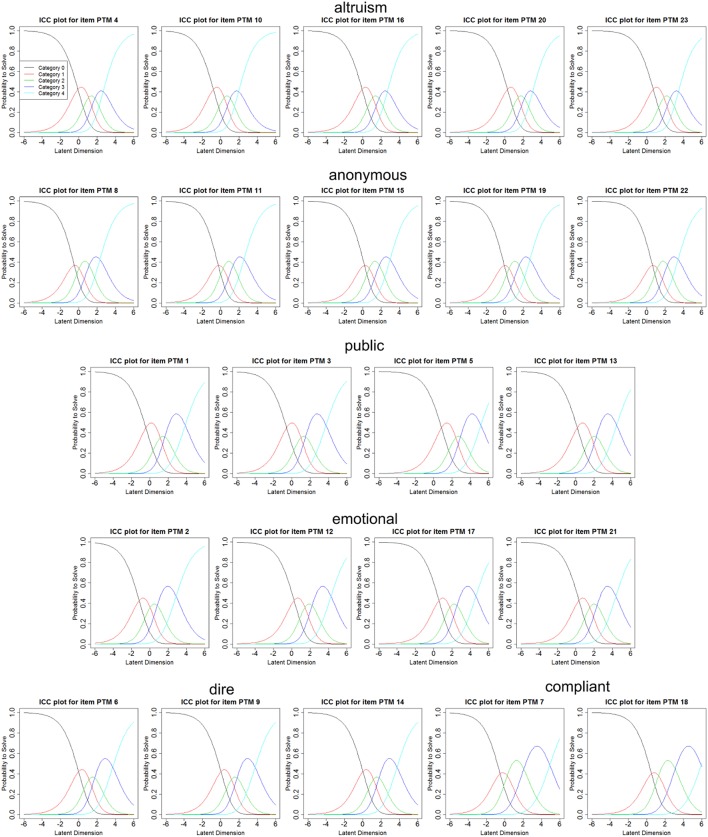
Item characteristic curves for the all items of the PTM-R.

The results of the item characteristics can be seen in Figure 2 and Table 2. The results show good item characteristics for every question as every answer category shows a distinct peak of probability at some point of the continuum where it is the most probable answer category to be picked None of these probability peaks were overpowered by other answer categories. Also a good differentiation of the item difficulty is given.

**Table 2:** Item difficulty and intersections of the item response curves of the rating scale model for the German version of the PTM-R.

**Items**	**Item number PTM-R**	**location IRT (difficulty)**	**Intersection 1 IRT**	**Intersection 2 IRT**	**Intersection 3 IRT**	**Intersection 4 IRT**
altruism1	4	1.35	−0.10	1.07	1.76	2.66
altruism2	10	0.64	−0.81	0.36	1.05	1.94
altruism3	16	1.37	−0.08	1.10	1.78	2.68
altruism4	20	1.74	0.29	1.46	2.15	3.05
altruism5	23	2.15	0.70	1.87	2.56	3.46
anonymous1	8	0.75	−0.49	0.03	1.14	2.31
anonymous2	11	0.96	−0.27	0.25	1.35	2.52
anonymous3	15	1.42	0.18	0.70	1.81	2.98
anonymous4	19	1.18	−0.05	0.47	1.57	2.74
anonymous5	22	1.86	0.62	1.14	2.25	3.42
public1	1	1.56	−0.43	1.14	1.64	3.87
public2	3	1.42	−0.57	1.00	1.50	3.73
public3	5	2.83	0.84	2.41	2.91	5.15
public4	13	2.15	0.16	1.73	2.23	4.46
emotional1	2	0.64	−1.16	0.03	0.81	2.88
emotional2	12	2.05	0.25	1.44	2.22	4.28
emotional3	17	2.36	0.56	1.76	2.53	4.60
emotional4	21	2.15	0.35	1.54	2.32	4.39
dire1	6	1.63	−0.03	1.13	1.74	3.70
dire2	9	1.68	0.02	1.17	1.78	3.75
dire3	14	1.68	0.01	1.17	1.78	3.74
compliant1	7	1.69	−0.51	0.26	2.08	4.92
compliant2	18	2.71	0.51	1.29	3.10	5.94

*Sample size is 534*.

For the confirmatory factor analysis, the fit indices for the target model reported by onyx software were: *X*^2^_*model(238)*_ = 542.38, *p* < 0.001; *RMSEA* = 0.053; *CFI* = 0.903; *TLI* = 0.886; *SRMR* = 0.058. Fit indices for the model in lavaan were identical except for *CFI* = 0.910, *TLI* = 0.904, *SRMR* = 0.0561, and *RMSEA* = 0.049 with 90% confidence interval 0.044–0.054. *TLI* and *CFI* did not support the model proposed, but *RMSEA* and *SRMR* indicate that this model had a good fit. The *X*^2^ did also not support the model, however it can be argued, that the sample size was very high, so “trivial differences between the sample and the estimated population covariance matrices are often significant because the minimum of the function is multiplied by *N*-1” (Tabachnick and Fidell, 2007, p. 715). The comparison with the one-factor solution led to a significant difference in *X*^2^: *t*_(15)_ = 1,720.8, *p* < 0.001, with the target model having a better fit and *X*^2^. Also, *AIC*_*six*−*factor model*_ = 31,809 compared to *AIC*_*one*−*factor model*_ = 33,500 and *BIC*_*six*−*factor model*_ = 32,070 compared to *BIC*_*one*−*factor model*_ = 33,697.

**Figure 3:**
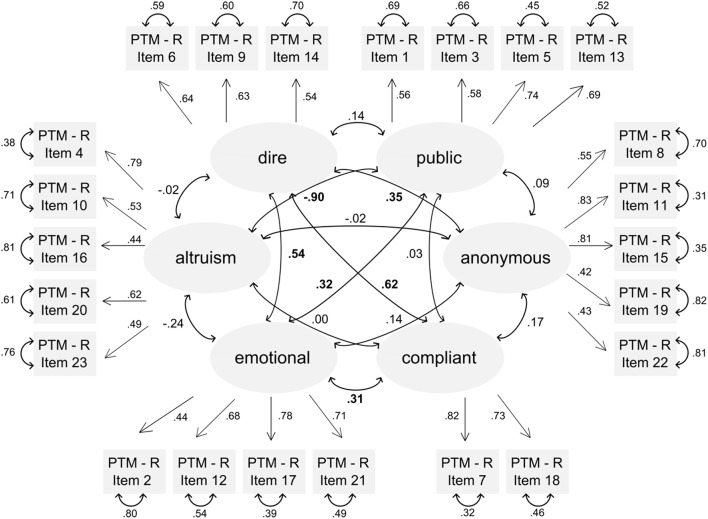
Model of confirmatory factor analysis, showing all six subcategories of prosocial behavior, their estimated correlations and the estimated relation to the questions of the translated version of the PTM-R. Bold marked correlations are at least medium size.

The model of the confirmatory factor analysis is shown in Figure 3. The estimation of the correlations of the latent variables can be seen in Figure 3 and in Table ESM1, containing the estimation of the correlation and the standard error of this estimation. For the latent variable of public prosocial behavior, correlations with altruism (*r* = −0.90) and emotional prosocial behavior (*r* = 0.32) were observed. While correlations between public prosocial behavior and altruism have also been observed for the original version of the PTM-R (Carlo et al., 2003), a relation to emotional prosocial behavior had not been reported so far. The positive correlations between dire and emotional prosocial behavior (*r* = 0.54), compliant and dire prosocial behavior (*r* = 0.62) and compliant and emotional prosocial behavior (*r* = 0.31) were in line with the findings of the original version of the PTM-R again. Also, for altruism, as in the original version, there was no correlation with anonymous and dire prosocial behavior. For the rest of the latent factors, there was a negligible or weak positive correlation, as could be also found in the original English version of the questionnaire, with the exception that anonymous and dire prosocial behavior showed a moderate positive correlation (*r* = 0.35).

Measurement invariance to the original English dataset was determined via Δ-CFI. For the metric or factor loading invariance Δ-CFI = 0.007 and Δ-RMSEA = 0.001. Hence metric invariance was given following Chen (2007) and Cheung and Rensvold (2002). This means that the factors of both samples had the same unit or same interval. For the intercept invariance or scalar invariance Δ-CFI = 0.002 and Δ-RMSEA = 0.002. Thus the scalar invariance was given following Chen (2007), Cheung and Rensvold (2002), and even Meade et al. (2008). This means that the scores of the different language groups had the same unit of measurement and the same intercept and therefore origin. Hence one can compare latent mean differences across groups (Widaman and Reise, 1997). For residual invariance Δ-CFI = 0.008 and Δ-RMSEA = 0.001. So, residual invariance was present following Chen (2007) and Cheung and Rensvold (2002). This invariance meant that all differences on the items are due to the group differences on the common latent factors.

### Subset 1: empathy, cognitive sensitivity, emotional sensitivity, emotional concern, cognitive concern, and social desirability

The means, standard deviations, and ranges for the subscales of the PTM-R are presented in Table ESM1. Cronbach's alpha of the validating constructs empathy, cognitive sensitivity, emotional sensitivity, emotional concern, cognitive concern and social desirability can be seen in Table 3. The relation of the subscales of the PTM-R to the validating constructs can be seen in Table 4. There were moderate correlations between empathy and the subscales of the E-Scales for empathy with dire prosocial behavior, but the correlation pattern was more pronounced for the emotional prosocial behavior subscale of the PTM-R. Social desirability correlated moderately with altruism and compliant prosocial behavior, as well as moderately negative with public prosocial behavior.

**Table 3:** Cronbach's alpha and McDonald's omega for validating constructs.

**Construct**	**Number of items**	**Cronbach's alpha subset 1**	**Cronbach's alpha subset 2**	**Cronbach's alpha subset 3**	**McDonald's omega subset 1**	**McDonald's omega subset 2**	**McDonald's omega subset 3**
Cognitive sensitivity	5	0.88	–	–	0.88 (0.86–0.90)		
Emotional sensitivity	6	0.64	–	–	0.79 (0.75–0.83)		
Emotional concern	7	0.79	–	–	0.80 (0.76–0.84)		
Cognitive concern	5	0.70	–	–	0.70 (0.64–0.75)		
Empathy total score	25	0.90	–	–	0.90 (0.88–0.92)		
SDS-CM	23	0.70	–	–	0.70 (0.63–0.75)		
Positive affect	10	–	0.85	–		0.85 (0.82–0.88)	
Negative affect	10	–	0.88	–		0.88 (0.85–0.90)	
Neuroticism	12^a^/20^b^	–	0.85	0.92		0.86 (0.83–0.88)	0.92 (0.90–0.93)
Extraversion	12^a^/20^b^	–	0.80	0.88		0.80 (0.76–0.84)	0.88 (0.85–0.90)
Openness	12^a^/20^b^	–	0.75	0.80		0.76 (0.72–0.80)	0.73 (0.63–0.79)
Agreeableness	12^a^/20^b^	–	0.75	0.80		0.76 (0.71–0.80)	0.77 (0.71–0.82)
Conscientiousness	12^a^/20^b^	–	0.84	0.83		0.84 (0.81–0.87)	0.83 (0.79–0.86)
Volatility	10	–	–	0.85			0.85 (0.81–0.88)
Withdrawal	10	–	–	0.89			0.67 (0.60–0.72)
Assertiveness	10	–	–	0.87			0.85 (0.82–0.87)
Enthusiasm	10	–	–	0.82			0.74 (0.68–0.79)
Intellect	10	–	–	0.82			0.88 (0.85–0.90)
Openness	10	–	–	0.76			0.83 (0.79–0.86)
Compassion	10	–	–	0.85			0.86 (0.83–0.88)
Politeness	10	–	–	0.68			0.90 (0.88–0.91)
Industriousness	10	–	–	0.84			0.82 (0.79–0.86)
Orderliness	10	–	–	0.73			0.76 (0.71–0.81)

a *Measured with NEO-FFI in subset 2*,

b *Measured with big five aspect scales in subset 3. Sample size of subset 1 is 285, sample size of subset 2 is 360, sample size of subset 3 is 326. In brackets, the 95%-confidence interval is reported*.

**Table 4:** Correlations of the subscales of PTM-R with E-Scales (empathy) and subscales of the E-Scales (cognitive sensitivity, emotional sensitivity, emotional concern, cognitive concern) and Crown-Marlow Social Desirability Scales (SDS -CM) in subset 1.

**Construct**	**Altruism**	**Anonymous**	**Public**	**Emotional**	**Dire**	**Compliant**
Cognitive sensitivity	−0.10	0.11	0.22^*^	0.40^**^	0.27^**^	0.13
Emotional sensitivity	0.01	0.004	0.08	0.38^**^	0.22^**^	0.14
Emotional concern	0.10	0.24^**^	0	0.44^**^	0.29^**^	0.23^**^
Cognitive concern	0.02	0.21^*^	0.07	0.46^**^	0.28^**^	0.17
Empathy	0.01	0.20	0.11	0.53^**^	0.34^**^	0.21^*^
SDS-CM	0.32^**^	0.04	−0.20^*^	−0.05	0.07	0.29^**^

* *p < 0.05*;

** *p < 0.01, Significance levels have been Bonferroni adjusted. Sample size is 285*.

### Subset 2: big five personality traits and positive and negative affect

The means, standard deviations, and ranges for the subscales of the PTM-R are presented in Table ESM1. In sum, the reported altruistic behavior confirmed findings of the entire dataset. The relation to other constructs like neuroticism, extraversion, openness, agreeableness and conscientiousness, as well as trait positive and negative affect can be seen in Table 5. Negative affect was negatively related to altruism. As hypothesized, neuroticism and extraversion were both positively related to emotional prosocial behavior. Finally, also as expected, agreeableness was correlated positively with altruism and also mildly positive with compliant prosocial behavior, as well as negatively with public prosocial behavior.

**Table 5:** Correlations of the subscales of PTM-R with trait positive and trait negative affect (measured via PANAS), neuroticism, extraversion, openness, agreeableness, and conscientiousness (measured via NEO-FFI) in subset 2.

**Construct**	**Altruism**	**Anonymous**	**Public**	**Emotional**	**Dire**	**Compliant**
Positive affect	−0.04	0.10	0.09	−0.02	0.18	0.05
Negative affect	−0.20^*^	0.00	0.19	0.13	−0.03	0.02
Neuroticism	−0.01	−0.06	−0.04	0.26^**^	−0.01	0.09
Extraversion	0.03	0.01	0.09	0.20^*^	0.18	0.06
Openness	0.15	0.10	−0.11	0.11	0.11	0.04
Agreeableness	0.39^**^	−0.05	−0.26^**^	0.04	0.08	0.19^*^
Conscientiousness	0.08	0.05	−0.02	−0.07	0.11	0.15

* *p < 0.05*;

** *p < 0.01, Significance levels have been Bonferroni adjusted. Sample size is 360*.

### Subset 3: big five personality traits and their aspects and a one shot dictator game

The means, standard deviations, and ranges for the subscales of the PTM-R are presented in Table ESM1. Reported prosocial behavior confirmed findings of the complete dataset. The relation to other constructs like neuroticism, extraversion, openness, agreeableness and conscientiousness can be seen in Table 6. As hypothesized above, neuroticism was positively correlated to emotional prosocial behavior, but this relation was likely due to the aspect withdrawal. Also according to our hypotheses, agreeableness had a strong positive relation with altruism and compliant prosocial behavior but also a lesser correlation with emotional prosocial behavior. Additionally, there was a negative correlation between public prosocial behavior and agreeableness. A similar pattern could be seen on the aspect compassion, where altruism, dire prosocial behavior, emotional prosocial behavior and compliant prosocial behavior were positively correlated. For the other aspect of agreeableness, the politeness, altruism and compliant prosocial behavior were positive correlated, but public prosocial behavior was negatively correlated. For extraversion, openness and conscientiousness, unexpectedly, there were no correlations with prosocial behavior tendencies. As well, there were no relations of their aspects to the prosocial behavior tendencies measured with the PTM-R. The Bonferroni adjusted correlation of the amount offered in the one-shot dictator game with the different subscales of the PTM-R are shown in Table 7.

**Table 6:** Correlations of the subscales of PTM-R with neuroticism, extraversion, openness, agreeableness, and conscientiousness (measured via Big-Five-Aspect-Scales) and the big five aspects volatility, withdrawal, assertiveness, enthusiasm, intellect, openness, compassion, politeness, industriousness, orderliness in subset 3.

**Construct**	**Altruism**	**Anonymous**	**Public**	**Emotional**	**Dire**	**Compliant**
Neuroticism	−0.21	0.13	0.12	0.28^**^	−0.04	−0.02
Extraversion	0.03	−0.06	0.03	0.06	0.17	0.09
Openness	0.19	0.10	−0.15	0.03	0.14	0.07
Agreeableness	0.43^**^	0.12	−0.31^**^	0.27^**^	0.21	0.40^**^
Conscientiousness	0.04	0.02	−0.02	−0.03	0.05	−0.10
Volatility	−0.19	0.07	0.12	0.23^*^	−0.01	−0.07
Withdrawal	−0.18	0.16	0.09	0.28^**^	−0.05	0.03
Assertiveness	−0.03	−0.05	0.04	−0.04	0.17	−0.04
Enthusiasm	0.08	−0.05	0.02	0.15	0.11	0.19
Intellect	0.14	0.01	−0.09	−0.12	0.08	−0.02
Openness	0.16	0.14	−0.15	0.17	0.14	0.12
Compassion	0.32^**^	0.08	−0.17	0.36^**^	0.33^**^	0.38^**^
Politeness	0.37^**^	0.11	−0.33^**^	0.07	0.01	0.26^**^
Industriousness	0.11	0.01	−0.08	−0.14	0.08	−0.08
Orderliness	−0.05	0.02	0.06	0.11	−0.02.	−0.09

* *p < 0.05*;

** *p < 0.01, Significance levels have been Bonferroni adjusted*.

*Sample size is 326*.

**Table 7:** Correlations of the subscales of PTM-R with the amount of money offered in a one shot dictator game in subset 3.

**Prosocial behavior tendency**	**Altruism**	**Anonymous**	**Public**	**Emotional**	**Dire**	**Compliant**
Amount offered in the one-shot dictator game	0.25^**^	0.04	−0.14	0.06	0.12	0.19^**^

** *p < 0.01, Significance levels have been Bonferroni adjusted. Sample size is 326*.

## Study II validation

In the study of Rodrigues et al. (2015), where the neuronal correlates of altruism in a dictator game were investigated, the PTM-R was used to assess the altruism of the participants (for details see Rodrigues et al., 2015). Also, as the complete questionnaire was used in this study to assess different prosocial tendencies, differences in the correlation of prosocial behavior in a dictator game and the different prosocial behavior tendencies assessed with the PTM-R can be shown. Additionally, the participants were asked whether they are engaged or were engaged in volunteer work or voluntary office activities. These results are reported here to validate the significance of the PTM-R and the subscales provided by this measure to prosocial behavior tendencies in real life situations or in observable prosocial behavior in economic games.

### Participants study II

40 participants (20 females *mean age* = 23.325, *SD* = 3.422, *range* = 19–31) participated in this study (for further details see Rodrigues et al., 2015). The sample is a subsample of subset 1 as mentioned above, where high and low scoring participants on the subscale altruism where selected.

### Paradigm study II

The paradigm used in this study was the dictator game (e.g., Kahneman et al., 1986; Güth, 1995; Haselhuhn and Mellers, 2005; Mellers et al., 2010), where a proposer may distribute an amount of money between him-or herself and a receiver. The receiver has no opportunity to interact with the proposer. Hence the dictator may freely distribute or even keep the money to him- or herself. As there is no strategic reason to offer a receiver anything of the money at all, a higher contribution to the receiver is interpreted as an altruistic act in this economic game. Here, the participant played in the role of the proposer. Beside the anonymity of the interaction, the income level of the receiver was manipulated. For details to the paradigm (see Rodrigues et al., 2015).

### Data analytic approach study II

For the analysis of correlation between the different prosocial behavior tendencies and the altruistic behavior in this game, a correlation for every subscale of the PTM-R was made with the behavior as a dictator in the condition where the receiver had the highest income, as the influence of altruism was highest in this condition (for details see Rodrigues et al., 2015). For the analysis of the reported volunteer work or voluntary office activities, also a correlation between the different subscales of the PTM-R was computed.

### Results study II

The results for the correlation can be seen in Table 8. In sum, as expected. only altruism correlated significantly with the altruistic behavior in the dictator game, while other prosocial behavior tendencies showed no significant relation to this prosocial behavior, Concerning the reported volunteer work or voluntary office activities, significant negative relation could be seen for two prosocial behavior tendencies: Dire prosocial behavior, as we hypothesized, but also compliant prosocial behavior.

**Table 8:** Correlations (95%-Confidence intervals) of the subscales of PTM-R with the amount of money offered in a dictator game in the high income condition and with the reported volunteer work or honorary office activities.

**Prosocial behavior tendency**	**Altruism**	**Anonymous**	**Public**	**Emotional**	**Dire**	**Compliant**
Reported volunteer work or honorary office activities	0.02 (−0.18–0.20)	0.15 (−0.09–0.25)	0.01 (−0.18–0.20)	−0.09 (−0.24–0.13)	−0.34^*^ (−0.39 to −0.02)	−0.31^*^ (−0.38–0.0)
Amount offered in the dictator game, high income condition (see Rodrigues et al., 2015)	0.32^*^ (0.02–0.99)	0.17 (−0.22–0.72)	−0.23 (−0.87–0.14)	−0.23 (−0.83–0.14)	−0.08 (−0.67–0.40)	0.24 (−0.13–0.93)

* *p < 0.05, Sample size is 40*.

## Discussion

The PTM-R from Carlo et al. (2003), was translated into German and the psychometric properties were investigated in exploratory and confirmatory analyses. Also, measurement invariance was assessed comparing the German version of the PTM to the original English version of the PTM to ensure the generalizability of the theorized six-factor structure across two culture groups. In general, the results suggest that the six factors proposed by Carlo et al. were replicated. Moreover, there was evidence that the German language version of the PTM-R demonstrates numerous relations to theoretically-relevant constructs, which adds sources of validity evidence to the measure. Moreover, the findings support the notion that the six forms of prosocial behaviors are distinct; therefore, consistent with recent growing evidence on the importance of conceptualizing and operationalizing prosocial behavior as a multidimensional construct.

Confirmatory factor analysis accounting for the six-factor solution showed a good fit on the data in two of the reported fit indices. Remarkably, compared to another translation of the same measure, trying to fit confirmatory factor analysis on the construct (Azimpour et al., 2012), the fit indices are all rather well. The measurement invariance test showed invariance on factor loading level, intercept (origin) level and also on the residual level. Therefore, the factor structure of prosocial tendencies as proposed by Carlo et al. (2003) did overcome the language barrier and was replicated in German students. The present findings therefore provide support for the six different prosocial tendencies postulated and previously demonstrated (Carlo and Randall, 2002; Carlo et al., 2003). In confirmatory factor analysis, many expected relations between the different prosocial behavior types were found. Especially the very strong negative correlation between public prosocial behavior and altruism shows the complementarity of these two constructs, although they are distinct types of prosocial behavior. Also, the positive relations of the dire and emotional prosocial behavior as well as compliant and dire prosocial behavior are clearly shown in the model. These relations suggest that these types of prosocial behavior have a common basis.

Thus, if one takes into account the validating constructs that were used in the subsamples of the study, the common bases of certain prosocial behavior tendencies or constructs that differ between the different kinds of prosocial behavior can be discovered. One common basis of the subscales dire prosocial behavior, emotional prosocial behavior and compliant prosocial behavior (see Figure 2) could be empathy and subscales of empathy as can be seen in Table 4. These relations could also be shown for the English original of the PTM-R (Carlo et al., 2003). Also, one can find a correlation of the subscale altruism with social desirability in subset 1, indicating that the altruism scale may be confounded by a report bias in participants. Similar findings could already be revealed by Carlo et al. (2003) for the original version of the PTM-R.

Importantly, this study is also extending the view on the altruistic construct by taking into account the big five personality traits and their aspects as well as positive and negative emotionality. We found that altruism was moderately related to agreeableness, as well as moderately negatively related to negative affect. This indicates less altruistic acts from a person with more trait negative affect, or vice versa, that experiencing more negative affect predicts less altruism. The relation found for the aspects of agreeableness lead to an even more precise picture of altruism assessed with the PTM-R, as compassion seems to play an important role in this concept, but also politeness plays also an important role in altruism. Hence the altruistic behavior tendencies measured here are not only driven by compassion and empathy, but also by the need of being polite and therefore maybe some motivation to experience harmony in life.

The subscale public prosocial behavior, which is negatively correlated to altruism, is also negatively correlated to agreeableness, which may reflect the egoistic motivation behind the public helping acts. Therefore, public prosocial behavior seems to be more of an opposite construct to altruism, but still is a distinct prosocial behavior tendency. Looking at the aspects of agreeableness, one can see that the negative relation is significant for politeness. Hence this type of prosocial behavior, that is related to cognitive aspects of empathy (see subset 1), is executed if one does not have the need to be polite. This more rational approach to prosocial behavior is yet negatively related to social desirability, maybe also indicating a more rational choice making and utilitarianism (Mill, 2006) or preference utilitarianism (Singer, 2011) of the person who is acting in such manner.

Emotional prosocial behavior tendencies measured with the German version of the PTM-R are related positively to neuroticism, agreeableness and extraversion. This shows the necessity of having the sensitivity for the problems of others, denoted by neuroticism and the tendency to actually approach others in such emotional situations, showed by extraversion for emotional prosocial behavior. Additionally, this prosocial behavior category correlated with all subtypes of empathy (see subset 1), showing the high empathic drive of this type of prosocial behavior tendency.

Dire prosocial behavior, which also correlates with all empathy-related subscales measured in subset 1, however is just correlated with the aspect compassion. This fits the concept of dire prosocial behavior, because one can be motivated to help in a crisis by compassion. As no other aspect or concept is related with this kind of prosocial behavior, the motivation to show this behavior seems to be solely driven by the compassionateness.

The compliant prosocial behavior tendencies measured with the German version of the PTM-R, in contrast, are not related to all aspects of empathy, but only to emotional concern. Being also correlated with agreeableness, this shows the reciprocal and interactive nature of this prosocial behavior category. Looking at the aspects of agreeableness related to compliant prosocial behavior, one can see that compassion and politeness are correlated with this kind of prosocial behavior. This leads to the assumption, that there is a common basis for this kind of prosocial behavior and altruism, for they are sharing the same correlational pattern for the big five aspects measured with BFAS. Finally, the anonymous prosocial behavior is not correlated with any aspect of the big five personality aspects, but it is correlated with emotional and cognitive empathic concerns.

Regarding the correlations of the prosocial tendencies with other personality traits assessed in this study, one can identify empathy, cognitive and emotional concern, cognitive and emotional sensitivity and agreeableness as positive correlates of prosocial behavior. These findings fit the argument that empathy may be a driving force in some prosocial behavior categories (Carlo et al., 2003). However, there are some differences, distinguishing between the different types of prosocial behavior, mostly expressed by social desirability, neuroticism, extraversion and negative affect. Considering the results of the confirmatory factor analysis and the correlation of the subscales of the PTM-R with other constructs, the German version of the PTM-R replicates the findings of the original English language version, as well as extend them to a certain point, especially concerning the aspects of the BFAS. Furthermore, this study provides the opportunity for German speaking researchers to receive a validated instrument for the assessment of prosocial behavior.

### Validation via dictator game and volunteer work

Concerning the validation of the different subscales of the PTM-R, only a few differences are shown via experimental data in this study, but there are many more examples mentioned in other work (e.g., Carlo and Randall, 2002; Carlo, 2014; Carlo and Davis, 2016). In our work, in study II and in the subset 3, we showed that the altruism measured via the German version of the PTM-R is linked to offering more money in a dictator game (see Rodrigues et al., 2015 for further details), while other prosocial behavior tendencies are not predictive for this prosocial behavior, except compliant prosocial behavior tendency for the one-shot in the subset 3. As the offering of any money in the dictator game is seen as an altruistic act, and the altruism measured with the PTM-R is correlated with this behavioral measure of altruism while other prosocial behavior tendencies are not, the differentiation of the prosocial behavior tendencies seems useful. Also, concerning the reported volunteer work or voluntary office activities, we saw significant negative correlations with dire prosocial behavior as hypothesized, but also with compliant prosocial behavior. These two prosocial behavioral tendencies are mainly focused on certain situations that have to occur—a crisis in case of dire prosocial behavior and a previous helping behavior or the concrete chance of repayment of the own prosocial behavior for compliant prosocial behavior. Hence it is plausible, that these two prosocial behavior tendencies are not, or even negatively, related to a generally ongoing prosocial activity like volunteer work. As the other four subscales are not related to the volunteer work, and taking this together with the results concerning the altruism subscale and the offers in the dictator game, it is plausible that the different subscales of the PTM-R do assess different motives for prosocial behavioral tendencies and that it is important to differentiate between motives for prosocial behavior, as there are different predictions for different kinds of prosocial activities and behavior based on the motive that may drive this behavior.

### Limitations

One potential concern of the PTM-R is that all items are keyed in the same direction. Hence, it is possible, that response biases (e.g., individual differences in the tendency to agree (or disagree) with statements in general) could take an influence on the correlations within the items of the different prosocial tendency categories as well as on the correlations between the different prosocial tendency categories. So, for every prosocial behavior category, the Cronbachs' alpha is likely to be lower if reversed items would be used, as well as the correlations of the underlying constructs may be overestimated in some cases where positive correlations were found, but also underestimated when negative correlations were found. However, as there are similar relations of the subscales of the PTM-R that are highly correlated with each other with other constructs measured with less biased instruments (see Tables 4–6), the relations of the subscales of the German version of the PTM-R stated by the mere analysis of the PTM should be sound. Finally, another limitation is that the PTM-R is not covering all prosocial behavior tendencies that are possible in human interactions. But as this is a rather unrealistic goal for a short questionnaire, the authors still hope to provide a useful tool for the prosocial behavior tendencies that are assessed with this questionnaire.

The current study was designed as a first validation study of the German version of the PTM-R. Future studies might want to investigate the relation between the prosocial tendencies assessed by the PTM-R and the HEXACO personality model (Lee and Ashton, 2004). Similar to the big five model, it assesses the personality structure in general, but also includes three personality factors that seem especially relevant for altruistic tendencies (honesty-humility, agreeableness and emotionality, see Ashton et al., 2014). A German translation of the HEXACO questionnaire has been widely used in past studies (e.g., Moshagen et al., 2014) and could easily be combined with the PTM-R in future studies.

In summary, correlations of the different subscales of the German version of the prosocial behavior tendencies measure with different personality traits like empathy, agreeableness and social desirability are found, validating the six different constructs of prosocial behavior tendencies proposed by the original questionnaire (Carlo and Randall, 2002; Carlo et al., 2003). These six different subtypes of prosocial behavior were also linked to personality traits in order to define their differences and possible common correlates. Importantly, there was not a single factor or construct that could explain all the different types of prosocial behavior, as could also be shown in a validation via dictator game and assessment of volunteer work. However, because there were substantial correlations between personality traits previously linked to the different kinds of prosocial behavior (e.g., Carlo and Randall, 2002; Carlo et al., 2003; Ben-Ner and Kramer, 2011; Leliveld et al., 2012), the German translation of the PTM-R is a valid measure of these prosocial tendencies for German speaking subjects. Therefore, it is possible to make the necessary distinctions between the six different types of prosocial behavior with this instrument in German-speaking young adults.

## Author contributions

JR: Data collection (Germany), Translation of PTM-R, writing of manuscript, data preparation and analyses. NU: Translation and retranslation of PTM-R, correction of manuscript, conceptual advice. PM: Data collection (Germany), correction of manuscript, methodological and conceptual advice. GC: Author of PTM-R: support of translation, data collection (USA), correction of manuscript, conceptual advice and input. JH: Conceptual and methodological advice and input, correction of manuscript, translation of PTM-R.

### Conflict of interest statement

The authors declare that the research was conducted in the absence of any commercial or financial relationships that could be construed as a potential conflict of interest.

## Appendix

### German version of the PTM-R

Fragen: (Items)

Ich kann Andern am besten helfen, wenn man mir zuschaut.

Es gibt mir ein gutes Gefühl, wenn ich jemanden trösten kann, der sehr aufgebracht ist.

Wenn andere Menschen um mich sind, fällt es mir einfacher Bedürftigen zu helfen.

Ich denke, eines der besten Dinge daran anderen zu helfen ist, dass es mich gut aussehen lässt.

Ich habe am meisten davon Anderen zu helfen, wenn ich es vor anderen Menschen tue.

Ich tendiere dazu, Menschen zu helfen, die wirklich bedürftig sind oder eine Krise erleben. Wenn mich andere Menschen nach Hilfe fragen, zögere ich nicht zu helfen.

Ich ziehe es vor dann zu spenden, wenn niemand etwas davon weiß.

Ich tendiere dazu Menschen zu helfen, die schwer verletzt wurden.

Ich glaube, dass das Spenden von Gütern oder Geld dann am meisten bringt, wenn ich einen Vorteil daraus ziehe.

Ich tendiere dazu Bedürftigen zu helfen, wenn sie nicht wissen, wer ihnen geholfen hat.

Ich tendiere dazu anderen zu helfen, vor allem wenn sie sehr emotional sind.

Ich erbringe Höchstleistungen, wenn ich dabei beobachtet werde, wie ich anderen helfe.

Es ist leicht für mich anderen zu helfen, wenn sie sich in einer schwierigen Situation befinden.

Meistens helfe ich anderen, wenn sie nicht wissen, wer ihnen geholfen hat.

Ich glaube ich sollte mehr entlohnt werden für die Zeit und Energie, die ich in ein Ehrenamt investiere.

Ich reagiere am besten auf ein Hilfegesuch, wenn die Situation sehr emotional ist.

Ich helfe anderen immer sofort, wenn sie nach Hilfe fragen.

Ich denke, anderen zu helfen ohne sie zu kennen ist die schönste aller Situationen.

Eines der schönsten Dinge an Ehrenamt ist es, dass es sich gut in meinem Lebenslauf liest.

Emotionale Situationen führen dazu, dass ich Bedürftigen helfen möchte.

Ich spende oft für etwas ohne den Empfänger zu kennen, da es mir ein gutes Gefühl gibt.

Ich bin der Meinung, dass wenn ich jemandem helfe, er mir in Zukunft helfen sollte.

[Ich helfe oft, sogar wenn ich der Meinung bin, dass ich nichts davon haben werde.]

[Ich helfe anderen normalerweise, wenn sie sehr aufgebracht sind.]

Antwortskala: (Scale)

Beschreibt mich gar nicht, Beschreibt mich ein wenig, Beschreibt mich einigermaßen, Beschreibt mich ganz gut, Beschreibt mich sehr gut.

Instruktionen allgemein zu Beginn der Fragebögen: (Instructions at the beginning) Dieser Fragebogen umfasst einige Aussagen, welche sich zur Beschreibung Ihrer eigenen Person eignen könnten.

Lesen Sie bitte jede dieser Aussagen aufmerksam durch und überlegen Sie, ob diese Aussage auf Sie persönlich zutrifft oder nicht.

Zur Bewertung jeder Aussage steht Ihnen eine fünffach abgestufte Skala zur Verfügung.

Es gibt bei diesem Fragebogen keine, richtigen ‘oder, falschen’ Antworten, und Sie müssen kein Experte (keine Expertin) sein, um den Fragebogen angemessen beantworten zu können. Sie erfüllen den Zweck der Befragung am besten, wenn Sie die Fragen so wahrheitsgemäß wie möglich beantworten.

Bitte lesen Sie jede Aussage genau durch und wählen Sie als Antwort die Kategorie an, die Ihre Sichtweise am besten ausdrückt. Bitte bewerten Sie die Aussagen zügig aber sorgfältig. Lassen Sie keine Aussage aus. Auch wenn Ihnen einmal die Entscheidung schwerfallen sollte, wählen Sie trotzdem immer eine Antwort, und zwar die, welche noch am ehesten auf Sie zutrifft.

Instruktionen spezifisch: (Instructions 2)

Im Folgenden sehen Sie ein paar Aussagen, die Sie mehr oder weniger gut beschreiben. Bitte geben Sie an, wie gut jede Aussage Sie beschreibt, indem sie die folgende Skala benutzen:

1 (Beschreibt mich gar nicht), 2 (Beschreibt mich ein wenig), 3 (Beschreibt mich einigermaßen), 4 (Beschreibt mich ganz gut), 5 (Beschreibt mich sehr gut).
