# Supplementary material for: Measuring Prosocial Tendencies in Germany: Sources of Validity and Reliablity of the Revised Prosocial Tendency Measure
Source: Front Psychol. 2017 Dec 6;8:2119. doi: 10.3389/fpsyg.2017.02119 (PMC5723663; doi:10.3389/fpsyg.2017.02119)
Supplement: Supplementary file 1 [file Presentation1.ZIP › ESM/Statistics/R/Output_Factor_Analysis_1.pdf]

RKWard output initialized on Mon Jan 30 11:29:53 2017

Import SPSS data

Parameters

- File name: F:/Documents/NACHSTUDIUM/STUDIEN/ALTRUISMS_SAMMLUNG/PTM-R_daten/Daten_PTM_R_fuer_R_kward_factor_odd_sample.sav
- Object to save to: my.spss.data

Mon Jan 30 11:31:32 2017

Messages, warnings, or errors:

Warning in read.spss("F:/Documents/NACHSTUDIUM/STUDIEN/ALTRUISMS_SAMMLUNG/PTM-R_daten/Daten_PTM_R_fuer_R_kward_factor_odd_sample.sav", :
F:/Documents/NACHSTUDIUM/STUDIEN/ALTRUISMS_SAMMLUNG/PTM-R_daten/Daten_PTM_R_fuer_R_kward_factor_odd_sample.sav: Unrecognized record type 7, subtype 18 encountered in system file

Run again

Parallel analysis (Horn) results

Mon Jan 30 11:32:32 2017

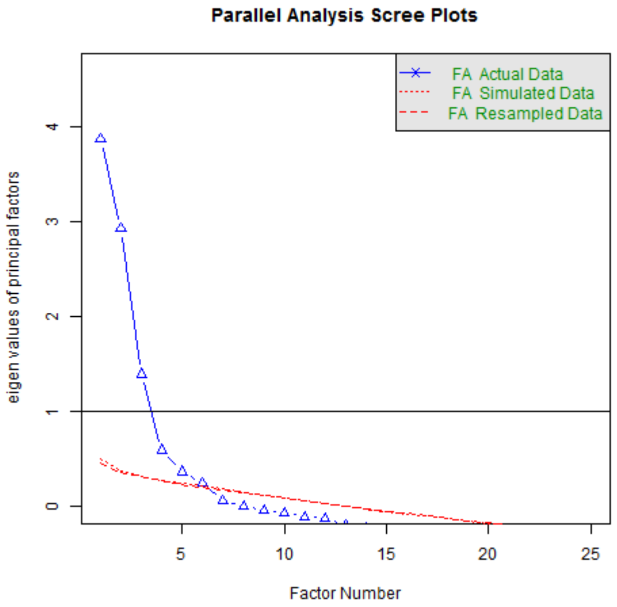

Messages, warnings, or errors:

Loading required package: psych
Warning: package 'psych' was built under R version 3.2.5

Parallel analysis suggests that the number of factors = 6 and the number of components = NA

Run again

Factor Analysis

Parameters

- Number of factors: 6
- Factoring method: minres
- Rotation: oblimin

Mon Jan 30 11:34:46 2017

Degrees of freedom	Fit	Fit (off diag)
165	0.842	0.989

Loadings

	Factor.1	Factor.2	Factor.3	Factor.4	Factor.5	Factor.6	communality	uniqueness
PTM_R1	0.075	0.044	**0.564**	0.045	-0.010	-0.023	0.316	0.684
PTM_R2	**0.333**	-0.064	**0.114**	**0.178**	**0.268**	0.011	0.294	0.706
PTM_R3	0.061	0.047	**0.728**	**0.160**	-0.016	-0.010	0.459	0.541
PTM_R4	0.047	0.082	-0.538	**0.272**	-0.003	0.085	0.532	0.468
PTM_R5	-0.059	-0.026	**0.598**	-0.311	-0.049	-0.016	0.644	0.356
PTM_R6	0.035	-0.010	-0.005	0.019	**0.760**	-0.076	0.566	0.434

PTM_R7	-0.043	-0.032	0.010	**0.128**	**0.270**	**0.541**	0.488	0.512
PTM_R8	-0.063	**0.561**	-0.144	-0.056	0.072	0.024	0.349	0.651
PTM_R9	-0.006	**0.145**	0.078	-0.012	**0.502**	0.050	0.328	0.672
PTM_R10	0.003	-0.049	-0.128	**0.614**	0.061	**0.102**	0.523	0.477
PTM_R11	0.001	**0.844**	0.025	0.067	-0.022	-0.044	0.707	0.293
PTM_R12	**0.766**	0.024	0.047	0.084	0.009	0.011	0.614	0.386
PTM_R13	-0.005	-0.043	**0.570**	-0.155	0.092	**0.149**	0.452	0.548
PTM_R14	**0.135**	0.073	-0.093	-0.106	**0.468**	**0.187**	0.417	0.583
PTM_R15	0.006	**0.789**	-0.003	-0.078	0.011	-0.009	0.628	0.372
PTM_R16	0.041	-0.034	-0.039	**0.488**	-0.085	-0.020	0.246	0.754
PTM_R17	**0.821**	-0.021	-0.032	-0.088	-0.060	-0.013	0.635	0.365
PTM_R18	0.045	-0.018	0.003	-0.013	-0.083	**0.838**	0.682	0.318
PTM_R19	**0.127**	**0.386**	0.087	0.003	0.023	**0.242**	0.292	0.708
PTM_R20	-0.055	-0.008	-0.045	**0.628**	0.029	-0.032	0.431	0.569
PTM_R21	**0.606**	0.091	0.016	-0.031	0.086	0.064	0.487	0.513
PTM_R22	0.080	**0.406**	0.094	-0.063	0.055	0.047	0.222	0.778
PTM_R23	-0.176	0.065	-0.040	**0.482**	-0.041	0.086	0.312	0.688
PTM_R24	0.021	**0.156**	-0.031	0.094	**0.301**	**0.290**	0.332	0.668
PTM_R25	**0.498**	-0.080	-0.023	-0.043	**0.217**	0.053	0.397	0.603
Sum of squared loadings	2.282	2.112	2.114	1.790	1.642	1.414	11.355	
Variance explained (%)	9.128	8.448	8.456	7.162	6.570	5.655		
Variance explained (cum %)	9.128	17.576	26.032	33.194	39.763	45.419		

Factor correlations

	Factor.1	Factor.2	Factor.3	Factor.4	Factor.5	Factor.6
Factor 1	1.000	0.145	0.161	-0.064	0.417	0.352
Factor 2	0.145	1.000	-0.015	0.042	0.237	0.069
Factor 3	0.161	-0.015	1.000	-0.489	-0.027	-0.038
Factor 4	-0.064	0.042	-0.489	1.000	0.151	0.194
Factor 5	0.417	0.237	-0.027	0.151	1.000	0.340
Factor 6	0.352	0.069	-0.038	0.194	0.340	1.000

Measures of factor score adequacy

	Factor 1	Factor 2	Factor 3	Factor 4	Factor 5	Factor 6
Correlation of scores with factors	0.915	0.916	0.897	0.874	0.866	0.880
Multiple R square of scores with factors	0.838	0.840	0.805	0.764	0.750	0.774
Minimum correlation of possible factor scores	0.676	0.679	0.611	0.528	0.[illegible]	[illegible]

Show TOC Go to top

Messages, warnings, or errors:

Loading required namespace: GPArotation
Warning in rk.header("Factor Analysis", parameters = list("Number of factors", :
Unnamed parameter lists are deprecated in rk.header()

Run again

Parallel analysis (Horn) results

Mon Feb 06 14:19:26 2017

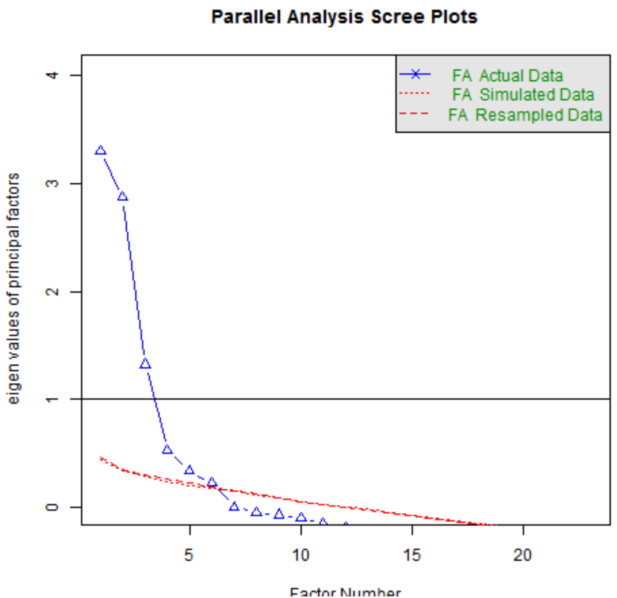

Messages, warnings, or errors:

Loading required package: psych
Warning: package 'psych' was built under R version 3.2.5

Parallel analysis suggests that the number of factors = 6 and the number of components = NA

Run again

Factor Analysis

Parameters

- Number of factors: 6
- Factoring method: minres
- Rotation: oblimin

Mon Feb 06 14:20:10 2017

Degrees of freedom	Fit	Fit (off diag)
130	0.839	0.990

Loadings

	Factor.1	Factor.2	Factor.3	Factor.4	Factor.5	Factor.6	communality	uniqueness
PTM_R1	0.047	**0.567**	0.074	0.043	-0.027	-0.023	0.320	0.680
PTM_R2	-0.059	**0.121**	**0.318**	**0.180**	**0.264**	0.030	0.285	0.715
PTM_R3	0.043	**0.729**	0.063	**0.152**	-0.016	-0.015	0.464	0.536
PTM_R4	0.082	-0.526	0.052	**0.288**	0.002	0.079	0.531	0.469
PTM_R5	-0.030	**0.582**	-0.064	-0.330	-0.034	-0.014	0.639	0.361
PTM_R6	-0.013	-0.009	0.016	0.007	**0.816**	-0.064	0.646	0.354
PTM_R7	-0.027	0.008	-0.037	**0.132**	**0.281**	**0.533**	0.481	0.519
PTM_R8	**0.570**	-0.139	-0.068	-0.048	0.055	0.030	0.351	0.649
PTM_R9	**0.160**	0.080	-0.008	-0.008	**0.468**	0.075	0.307	0.693
PTM_R10	-0.046	-0.116	0.008	**0.624**	0.053	0.098	0.521	0.479
PTM_R11	**0.841**	0.027	-0.003	0.067	-0.013	-0.042	0.705	0.295
PTM_R12	0.017	0.051	**0.741**	0.083	0.028	0.017	0.591	0.409
PTM_R13	-0.040	**0.563**	0.001	-0.161	0.081	**0.149**	0.449	0.551
PTM_R14	0.081	-0.094	**0.148**	-0.097	**0.450**	**0.198**	0.407	0.593
PTM_R15	**0.790**	-0.002	0.007	-0.075	0.001	-0.008	0.627	0.373
PTM_R16	-0.036	-0.029	0.031	**0.490**	-0.073	-0.020	0.245	0.755
PTM_R17	-0.036	-0.037	**0.828**	-0.088	-0.037	-0.018	0.652	0.348
PTM_R18	-0.012	0.001	0.032	-0.011	-0.071	**0.856**	0.716	0.284
PTM_R19	**0.383**	0.083	**0.135**	0.004	0.026	**0.232**	0.288	0.712
PTM_R20	-0.006	-0.033	-0.059	**0.634**	0.025	-0.032	0.430	0.570
PTM_R21	0.084	0.015	**0.615**	-0.027	0.087	0.064	0.495	0.505
PTM_R22	**0.405**	0.093	0.087	-0.062	0.042	0.040	0.218	0.782
PTM_R23	0.067	-0.032	-0.179	**0.486**	-0.034	0.079	0.311	0.689
Sum of squared loadings	2.079	2.073	1.967	1.815	1.448	1.299	10.680	
Variance explained (%)	9.039	9.014	8.551	7.891	6.294	5.646		
Variance explained (cum %)	9.039	18.052	26.603	34.494	40.788	46.434		

Factor correlations

	Factor.1	Factor.2	Factor.3	Factor.4	Factor.5	Factor.6
Factor 1	1.000	-0.013	0.158	0.040	0.230	0.059
Factor 2	-0.013	1.000	0.168	-0.490	-0.020	-0.026
Factor 3	0.158	0.168	1.000	-0.064	0.397	0.347
Factor 4	0.040	-0.490	-0.064	1.000	0.150	0.187
Factor 5	0.230	-0.020	0.397	0.150	1.000	0.298
Factor 6	0.059	-0.026	0.347	0.187	0.298	1.000

Measures of factor score adequacy

	Factor 1	Factor 2	Factor 3	Factor 4	Factor 5	Factor 6
Correlation of scores with factors	0.915	0.895	0.908	0.876	0.872	0.885
Multiple R square of scores with factors	0.837	0.802	0.825	0.768	0.761	0.783
Minimum correlation of possible factor scores	0.675	0.604	0.650	0.536	0.521	0.565

Messages, warnings, or errors:

Loading required namespace: GPArotation
Warning in rk.header("Factor Analysis", parameters = list("Number of factors", :
  Unnamed parameter lists are deprecated in rk.header()

Run again
