# Supplementary material for: Measuring Prosocial Tendencies in Germany: Sources of Validity and Reliablity of the Revised Prosocial Tendency Measure
Source: Front Psychol. 2017 Dec 6;8:2119. doi: 10.3389/fpsyg.2017.02119 (PMC5723663; doi:10.3389/fpsyg.2017.02119)
Supplement: Supplementary file 1 [file Presentation1.ZIP › ESM/Statistics/R/Output_Factor_Analysis_2.html]

xml version="1.0" encoding="System"?

RKWard Output


```
RKWard output initialized on Mon Jul 31 18:00:28 2017
```

Hide TOC
Go to top
  
 
1 •
2 •
3 •
4

Show TOC
Go to top

# Parallel analysis (Horn) results

Mon Jul 31 18:00:41 2017  
  

## Messages, warnings, or errors:

```
Parallel analysis suggests that the number of factors =  6  and the number of components =  NA
```

Run again

---

# Factor Analysis

## Parameters

- Number of factors: 6
- Factoring method: minres
- Rotation: oblimin

Mon Jul 31 18:01:00 2017  
